# The SOS Response is Permitted in *Escherichia coli* Strains Deficient in the Expression of the *mazEF* Pathway

**DOI:** 10.1371/journal.pone.0114380

**Published:** 2014-12-03

**Authors:** Ziva Kalderon, Sathish Kumar, Hanna Engelberg-Kulka

**Affiliations:** Department of Microbiology and Molecular Genetics, IMRIC, The Hebrew University- Hadassah Medical School, Jerusalem, Israel; University of Manchester, United Kingdom

## Abstract

The *Escherichia coli* (*E. coli*) SOS response is the largest, most complex, and best characterized bacterial network induced by DNA damage. It is controlled by a complex network involving the RecA and LexA proteins. We have previously shown that the SOS response to DNA damage is inhibited by various elements involved in the expression of the *E. coli* toxin-antitoxin *mazEF* pathway. Since the *mazEF* module is present on the chromosomes of most *E. coli* strains, here we asked: Why is the SOS response found in so many *E. coli* strains? Is the *mazEF* module present but inactive in those strains? We examined three *E. coli* strains used for studies of the SOS response, strains AB1932, BW25113, and MG1655. We found that each of these strains is either missing or inhibiting one of several elements involved in the expression of the *mazEF*-mediated death pathway. Thus, the SOS response only takes place in *E. coli* cells in which one or more elements of the *E. coli* toxin-antitoxin module *mazEF* or its downstream pathway is not functioning.

## Introduction

The enteric bacterium *E. coli*, like most other bacteria, carries on its chromosome the gene pair *mazEF*, belonging to the abundant family of toxin-antitoxin modules [1]. *mazF* specifies for the stable toxin MazF [2], a sequence specific endoribonuclease, which cleaves at ACA sites [3]. *mazE* specifies for the labile antitoxin MazE, which is degraded by the protease ClpPA [2]. *E. coli mazEF* is responsible for bacterial programmed cell death (PCD) under stressful conditions [4]. Under such conditions, the induced endoribonuclease MazF removes the 3′-terminal 43 nucleotides of the 16S rRNA within the ribosomes, thereby removing the anti-Shine-Dalgarno (aSD) sequence that is required for translation initiation of canonical mRNAs. Concomitantly, MazF also cleaves at ACA sites at or closely upstream from the AUG start codon of certain specific mRNAs, causing the generation of leaderless mRNAs [5]. Thus, stressful conditions lead to the generation of the alternative translation machinery [5] which is responsible for the synthesis of stress proteins, some of which are involved in cell death and the others in cell survival [6]. Therefore, *mazEF* can be considered as a master regulatory element, that induces downstream pathway leading to the death of most of the population, and continued survival of a small subpopulation [6]. In addition, *E. coli mazEF-*mediated cell death is a population phenomenon requiring the participation of NNWNN, a linear penta-peptide, which is a quorum sensing factor that we have called the Extracellular Death Factor (EDF) [7]–[8]. EDF induces the enoribonucleolytic activity of *E. coli* MazF [9].

Recently, using confocal microscopy and FACS analysis we showed that under condition of sever DNA damage; the triggered EDF-*mazEF*-mediated cell death pathway leads to the inhibition of a second cell death pathway. The latter is an Apoptotic-Like Death that we have called ALD; ALD is mediated by *recA* and *lexA* [10]. The well known, extensively studied SOS pathway (reviewed by [11]–[15]) is also a cellular response to DNA damage, and is also mediated by *recA*-*lexA*. In an uninduced cell, the *lexA* gene product, LexA, acts as a repressor of more than 40 genes [16]–[17], including r*ecA* and *lexA*, by binding to operator sequences (called SOS box) upstream to each gene or operon. Under conditions of DNA damage, regions of single-stranded DNA are generated that convert RecA to an active form that facilitate an otherwise latent capacity of LexA (and some other proteins like UmuD and the λCI repressor) to auto digest [12], [14]–[16], [18]. We have recently shown that the *E. coli* EDF-*mazEF* pathway inhibits the SOS response as it inhibits the ALD pathway (19). Since the *mazEF* pathway is present on the chromosomes of most *E. coli* strains [20], [21], we asked why is the SOS response found in so many *E. coli* strains? Perhaps the EDF-*mazEF* pathway is present but not active in those strains?

## Results

### The Extra-Cellular Death Factor (EDF) is involved in the inhibition of the SOS response

**Figure 1:**
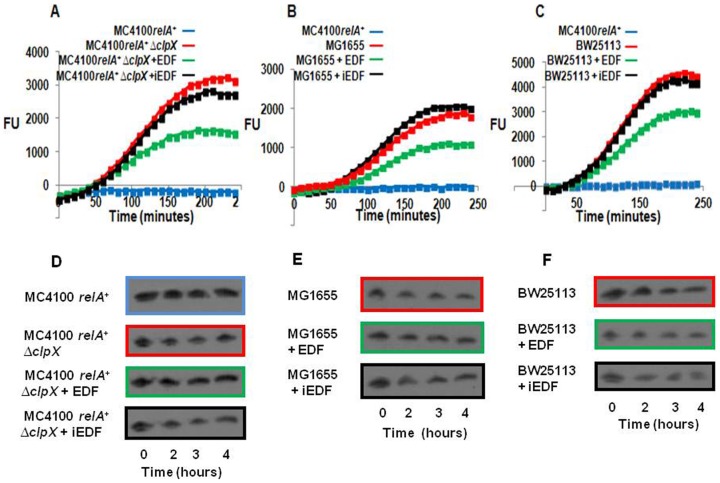
The inhibition of the SOS response by the *mazEF* pathway required the participation of EDF. We determined the SOS response by measuring the fluorescence of the reporter plasmid pL(*lexO*)-*gfp* (**A, B, C**), and by LexA degradation (**D, E, F**). We compared *E. coli* strain MC4100*relA* ^+^ (A and **D**) with strains MC4100*relA* ^+^Δ*clpX* (**A** and **D**), MG1655 (**B** and **E**), or BW25113 (**C** and **F**); the strains in A, B, and C harbored plasmid pL(*lexO*)-*gfp*. We grew the cells in M9 media supplemented with ampicillin (100 µg/ml), with shaking. When the culture reached O.D._600_ 0.5–0.6, we added (or not) EDF (10 ng/ml) or iEDF (100 ng/ml). These cultures were incubated without shaking at 37°C for 30 min, after which we added NA (10 µg/ml) to each sample. Immediately after adding NA, we measured fluorescence (FU) by fluorometer or LexA degradation (as described in Materials and Methods) over a period of 4 hours. The values shown are relative to those of cells that had not been treated with NA. All data are representative of three independent experiments. The colors surrounding the blots in D, E, and F correspond to the colors representing the samples in A, B, and C.

In previous studies we showed that EDF, the penta-peptide NNWNN, is involved in EDF-*mazEF* mediated cell death [7], and that *clpX* is required for the production of EDF [8]. Since, more recently we found that the action of the *mazEF* module prevented the SOS response [19]; here we asked if, in addition to the *mazEF* module, the presence of EDF is also involved in the inhibition of the SOS response. As previously [19], we also here studied the SOS response by the use of plasmid pL(*lexO*)-*gfp* [22], which bears the gene *gfp* under the control of the *lexA* operator, *lexO*. In this plasmid, under uninduced conditions, LexA represses *gfp* transcription by binding to the SOS box in the gene operator, *lexO*. Under DNA damage, RecA becomes activated, and acts as a co-protease stimulating the inactivation of LexA by auto-cleavage. Thus, in this system, fluorescence is a reporter for the RecA dependent SOS response. We caused DNA damage by adding nalidixic acid (NA) (10 µg/ml) to the cultures [19]. Our experiments have revealed that the SOS response was permitted not only in an *E. coli* MC4100*relA* ^+^ strain from which we deleted *mazEF* (MC4100*relA* ^+^Δ*mazEF*) [19], but also when, instead of deleting *mazEF*, we deleted *clpX* (MC4100*relA* ^+^Δ*clpX*) (Figure 1A). This effect seems to be due to the lack of EDF because: (a) the addition of EDF partially inhibits the studied SOS response (by 50%), and (b) the SOS response is not affected at all by the addition of iEDF (Figure 1A), the penta-peptide NNGNN, in which the central and crucial tryptophan has been replaced by glycine [8]. Adding iEDF to the MC4100*relA* ^+^Δ*clpX* culture did not affect the SOS response at all (Figure 1A). Similar results were obtained when instead of studying the SOS response by the use of plasmid pL(*lexO*)-*gfp*, we studied it by following the NA-induced LexA degradation (Figure 1D). Here, LexA degradation, thus SOS response is not permitted in *E. coli* MC4100*relA* ^+^ strain carrying the *mazEF* module (Figure1D, first line). However, deleting *clpX* (MC4100*relA* ^+^Δ*clpX*) permitted LexA degradation (Figure 1D, second line). On the other hand, the addition of EDF prevented LexA degradation (Figure 1D, third line), while LexA degradation is permitted by the addition of iEDF (Figure 1D, fourth line).

An additional support that EDF is involved in the *mazEF* mediated inhibition of the SOS response is derived from our studies with *E. coli* strain MG1655. In our previous work, we showed that *E. coli* strain MG1655, which carries the *mazEF* gene pair is defective in the production of and the response to EDF [8]. Here we found that, despite the presence of *mazEF*, the SOS response took place in strain MG1655 (Figure 1B). Furthermore, 240 minutes after adding EDF, we observed a 50% reduction in the SOS response; in contrast, adding iEDF did not cause any reduction in the SOS response (Figure 1B). Similar results were also obtained in *E. coli* strain MG1655 by studying the NA-induced LexA degradation (Figure 1E) under the SOS response condition. Also here, LexA degradation, thus the SOS response is permitted in *E. coli* MG1655 (Figure 1E, first line). On the other hand, the addition of EDF significantly prevented LexA degradation (Figure 1E, second line), while LexA degradation is again permitted by the addition of iEDF (Figure 1E, third line). All of these results support our hypothesis that the SOS response was permitted in the absence of EDF.

Using our fluorescence reporter system, we tested the SOS response in two additional *E. coli* strains. In strain AB1932 [23] the addition of EDF did not inhibit the SOS response (Figure S1). However, in *E. coli* strain BW25113, which has commonly been used to study the phenomena of the SOS response [23]–[24], the addition of EDF did reduce the SOS response (Figure 1C). Adding EDF to *E. coli* strain BW25113 led to a 30% reduction in the SOS response; again, as in the case for strains MC4100*relA*+Δ*clpX* (Figure 1A), and MG1655 (Figure 1B), adding iEDF did not lead to a reduction in the SOS response (Figure 1C). Similar results were obtained in *E.coli* strain BW25113 by determining the NA-induced LexA degradation. Also here, LexA degradation is permitted (Figure 1F, first line). However, its degradation is prevented by the addition of EDF (Figure 1F, second line), but not by the addition of iEDF (Figure 1F, third line). Thus, the SOS response is permitted in *E.coli* strains BW25113 and MG1655. This because of unknown reasons there is a deficiency of EDF in these strains (Table 1).

**Table 1:** The identified elements related to the EDF-*mazEF* pathway that permitted the SOS response in the herein studied *E. coli* strains.

Strains	The elements studied	
	EDF	λ lysogen
MG1655	[−]	[−]
BW25113	[−]	[−]
MC4100*relA* ^+^λ	[+]	[+]
AB1932λ	[+]	[+]

Brackets indicate the element that was missing [−] or present [+] that permitted the SOS response in each of these strains.

**Figure 2:**
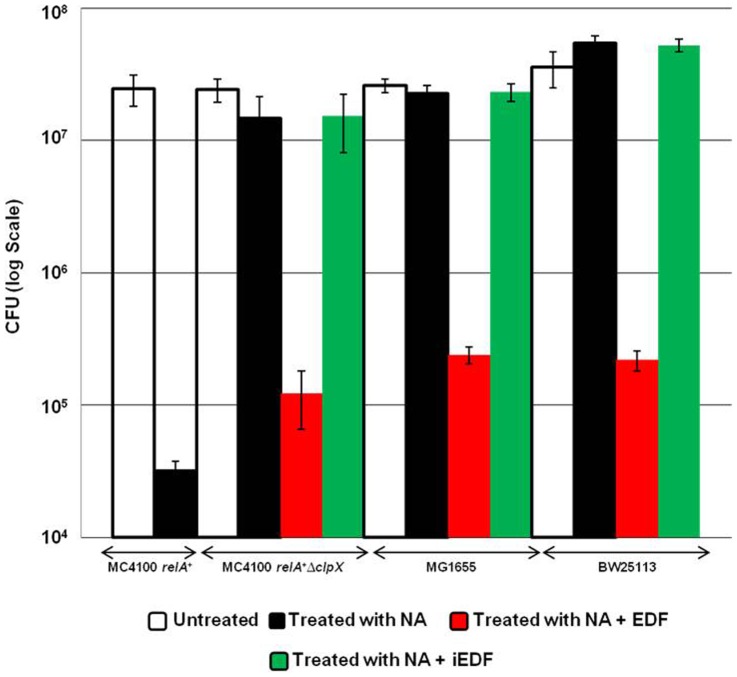
Cell viability is affected under DNA damage in *E.coli* strains and under conditions in which the SOS response is impaired. We studied the effect of cell viability at low concentrations of Nalidixic acid (10 µg/ml). *E. coli* strains WT MC4100*relA* ^+^ or MC4100*relA* ^+^Δ*clpX* or MG1655 or BW25113 were grown to OD_600_ 0.5. We divided each culture into aliquots to which we added no NA (untreated) or added NA 10 µg/ml (treated with NA) or added NA with EDF (treated with NA + EDF) or added NA with iEDF (treated with NA + iEDF) as described in the Legend to Figure 1. We carried out viability assays on LB plates as we have described previously (19).

Our findings suggesting that the SOS response is permitted in *E.coli* strains due to the lack of EDF is also manifested by testing the viability of these strains under conditions of DNA damage (Figure 2). In *E.coli* strain MC4100*relA* ^+^, in which the SOS responds is prevented (Figure 1A and 1D), cell viability is reduced by about 3 folds under such condition (Figure 2). In contrast, in the Δ*clpX* derivative of MC4100*relA*+ which is defective in the generation of EDF (Figure 1A and 1D), cell viability is not affected under the SOS conditions (Figure 2). However, by applying EDF cell survival is reduced by about 2 folds, and this reduction in viability does not occur by applying iEDF (Figure 2). A similar manifestation on the effect of DNA damage on cell viability was also observed in *E.coli* strains MG1655 and BW25113. These strains which are permitting the SOS response due to their lack of EDF (Figures 1B, 1E and Figure 1C, 1F, respectively), also permit cell survival under the SOS conditions (Figure 2). However, cell viability was reduced by about two folds when EDF was applied to these stains, and this reduction in viability does not occur by applying iEDF (Figure 2).

### The SOS response is permitted in *E. coli* strains carrying prophage lambda

**Figure 3:**
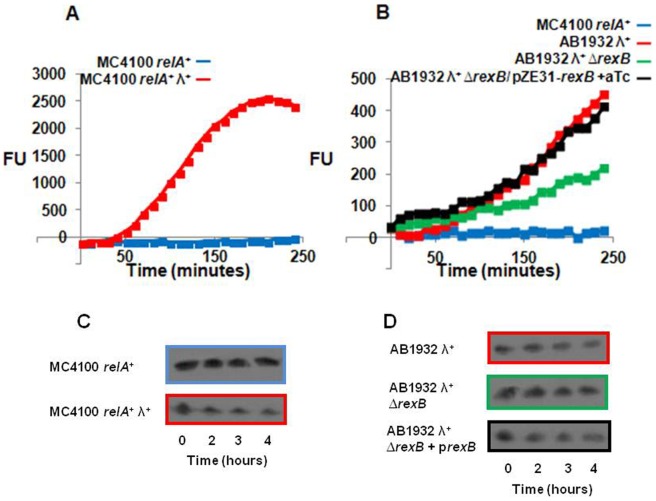
λ Lysogens overcome the inhibitory effect of *mazEF* on the SOS response. We determined the SOS response by measuring the fluorescence of the reporter plasmid pL(*lexO*)-*gfp* (**A, B**), and by LexA degradation (**C**, **D**). We used *E. coli* MC4100*relA* ^+^ as a control strain, and two experimental parent strains lysogenized by phage *λ*: MC4100*relA* ^+^ *λ* ^+^ (**A, C**), and AB1932*λ* ^+^, AB1932*λ* ^+^Δ*rexB*, AB1932*λ* ^+^Δ*rexB*/*pZA31-rexB* (**B, D**). The strains in A and B harbored plasmid pL(*lexO*)-*gfp*. Cells were grown as described in the Legend to Figure 1, except that the ampicillin concentration was (25 µg/ml), and chloramphenicol (6.25 µg/ml) was added to cells harboring plasmid pZE31-*rexB*. At O.D._600_ 0.5–0.6, the strains harboring plasmid pZE31-*rexB* were induced by the addition of aTc (0.5 µg/ml), and incubated without shaking at 37°C for 30 min after which we added NA (10 µg/ml). We measured fluorescence (FU) (**A** and **B**) by fluorometer or LexA degradation (**C** and **D**) over a period of 4 hours. The values shown are relative to those of cells not treated by NA. All data are representative of three independent experiments. The colors surrounding the gels in C and D correspond to the colors representing the samples in A and B.

One of the few genes expressed by phage λ in its lysogenic state is λ*rexB* [25]–[27]. In previous work, we showed that its product, λRexB, inhibits the degradation of the antitoxic labile compound, MazE, thereby preventing *mazF* mediated death pathway [28]. Therefore, we anticipated that, in contrast to *E. coli* strain MC4100*relA* ^+^ in which the SOS response is prevented (Figure 1A and 1D), in the presence of a λ prophage the SOS response would be permitted in this strain. As we expected, the presence of the λ prophage overcame the inhibitory effect of *mazEF* on the SOS response (Figure 3A). Similar results were obtained in *E. coli* strain MC4100*relA* ^+^ when instead of studying the SOS response by the use of plasmid pL(*lexO*)-*gfp*, we studied it by following LexA degradation (Figure 3C). Here, the NA-induced LexA degradation, thus the SOS response, is permitted in an *E. coli* strain carrying prophage λ, MC4100*relA* ^+^λ (Figure 3C, second line), and not in the absence of the prophage from the chromosome of this bacterial strain (Figure 3C, first line). Also, in *E.coli* strain AB1932, in which the SOS response has been observed [23], and which has been reported to bear a λ prophage on its chromosome [23], we observed the SOS response both by the use of plasmid pL(*lexO*)-*gfp* (Figure 3B) and by following LexA degradation (Figure 3D, first line). Furthermore, deleting *rexB* from its λ prophage reduced the SOS response by 50%, while introducing a plasmid bearing λ*rexB* and inducing it permitted the SOS response (Figure 3B). In addition, deleting *rexB* from its λ prophage stabilized LexA degradation (Figure 3D, second line), while introducing a plasmid bearing λ*rexB* and inducing it permitted LexA degradation (Figure 3D, third line), Thus, our results provide an explanation for the SOS response in strain AB1932λ (Table 1).

**Figure 4:**
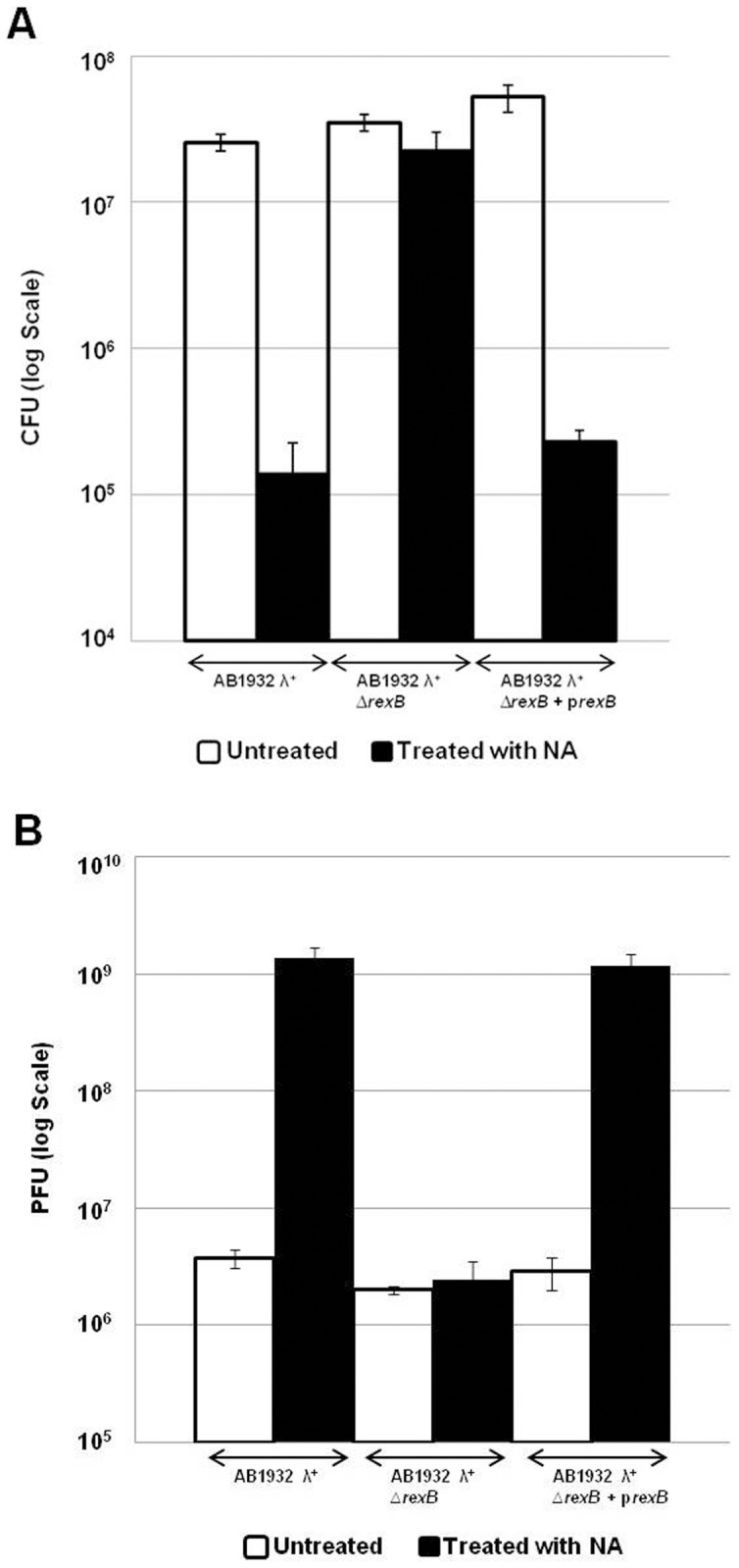
λ*rexB* is involved in the transfer from lysogenic to lytic stage. (**A**) Cell viability (CFU/ml) and (**B**) **λ** plaque forming units (PFU/ml). The *E. coli* strains AB1932*λ* ^+^ or AB1932*λ* ^+^Δ*rexB* or AB1932*λ* ^+^Δ*rexB*/*pZA31-rexB* were grown as described in the Legend to Figure 2. We carried out viability assays on LB plates as we have described previously (19) and the PFU were determined as described in Materials and Methods.

Our findings suggesting that the SOS response is permitted in an *E.coli* strain carrying prophage lambda was further supported by testing the viability of strain AB1932λ under conditions of DNA damage (Figure 4A). In this strain, in which we have shown that the SOS responds is permitted due to the presence of λ*rexB* gene (Figures 3B and 3D), We found that also cell viability is reduced by about two folds under conditions of DNA damage (Figure 4A). In contrast, in the Δ*rexB* derivative of strain AB1932λ in which the SOS response is severely affected (Figures 3B and 3D); cell viability is not affected under the SOS conditions (Figure 4A). However, introducing a plasmid bearing λ*rexB* and inducing it, lead to the reduction of cell viability by about two orders (Figure 4A).

Furthermore, as expected, the observed reduction in cell viability by applying DNA damaging conditions to *E.coli* strain AB1932 λ carrying the *rexB* gene (Figure 4A) is due to the transfer of the phage from its lysogenic to its lytic stage (Figure 4B). This is here shown by the determination of the plaque forming ability of the phage which we found to be 10^9^ PFU/ml when the λ lysogens carried *rexB* or when λ *rexB* was carried on a plasmid, and 10^6^ PFU/ml in the absence of λ*rexB* (Figure 4B). Such a result is an outcome of the process of the induction of prophage λ which is a classical manifestation of the SOS response (see under Discussion).

## Discussion

The bacterial response to DNA damage was first described by Radman in the 70′s, who called it the “SOS response” [13]. To date, it is the largest, most complex, and best understood bacterial DNA damage-inducible network to be characterized [11]–[15], [18]. The expression of genes in the SOS regulatory network is controlled by a complex circuit involving the RecA and LexA proteins [15]. In response to an SOS-inducing treatment or conditions, regions of single-stranded DNA are generated. The binding of RecA to these regions of single-stranded DNA in the presence of nucleotide triphosphate generates a nucleotide filament, and as a result converts RecA to an active form that facilitates an otherwise latent capacity of LexA (and some other proteins like UmuD and the λ repressor CI) to undergo self-cleavage [12], [14]–[16], [18].

We have previously shown that the EDF-*mazEF* mediated death pathway inhibits the SOS response (19). This inhibition was caused by the expression of *mazF* itself, as well as the genes *yfbU*, *slyD*, *yfiD*, *clpP* and *ygcR* (19) specifying for proteins acting in the death pathway downstream to *mazEF* [6]. Moreover, as we have shown here the extra-cellular death factor EDF, the peptide NNWNN, is also involved in the inhibition of the SOS response (Figure 1). These results suggested that *E. coli* strains commonly used in studies of the SOS response may be defective in the expression of the EDF-*mazEF* pathway. Here we studied the following *E. coli* strains used in SOS response studies: AB1932 [23], BW25113 [24], [29] and MG1655 [16], [23]. We found that although these strains are carrying the *mazEF* module in their chromosome (Figure S2), they were missing or inhibiting other elements of the EDF-*mazEF* mediated death pathway (summarized in Table 1). Strains BW25113 and MG1655 are defective in the production of EDF (Figure 1 and Table 1). The SOS response was also studied in *E.coli* strain MC4100*relA1* [22] which is defective in the production of the starvation signaling molecule ppGpp [30]. We have previously shown that this strain indeed permits the SOS response [19], probably because ppGpp triggers the *mazEF* pathway [2].

Finally, among the *E. coli* strains commonly used in studies of the SOS response, the most interesting strain studied here is AB1932. Our results indicate that this strain is defective in the *mazEF*-mediated pathway because of being a λ lysogen (Figure 3B, 3D and Table 1), that carries the *rexB* gene of the phage [23]. Moreover, strain MC4100*relA^+^* in which the SOS response is inhibited (Figure 3A and the first line of 3C), enabled the SOS response when lysogenised with phage λ carrying *rexB* (Figure 3A and second line of 3B). We have previously reported that λRexB, the product of the λ*rexB* gene, prevents the degradation of the antitoxin MazE [28]. Thus, it is expected that in the presence of λRexB, MazF activity is prevented and therefore the SOS response is permitted. Indeed, here we found that, in strain AB1932λ (Figure 3B and 3D), simply deleting the λ*rexB* gene significantly reduced the SOS response. Moreover, we found that λ*rexB* is involved in the transfer from the lysogenic to the lytic stage of the phage (Figure 4B). This result is a self evident outcome of the process of λ phage induction which is a classical manifestation of the SOS response. Under conditions of DNA damage, RecA is converted to an active form that facilitates an otherwise latent capacity of λCI repressor to auto digest [18]. Thereby λ phage can be transferred from its lysogenic to its lytic stage. Thus, the product of λ*rexB* gene that prevents the degradation of the antitoxin MazE [28], and thereby prevents MazF activity, permits in turn the SOS response (Figure 3), Therefore, λRexB is a crucial factor that enables the transfer of phage λ from its lysogenic to its lytic stage.

Furthermore, our herein described study, showing that the SOS response was discovered and is permitted due to the use of *E. coli* strains deficient in the expression of the EDF-*mazEF* mediated pathway, is a striking example for the reason of different experimental results obtained by the use of different bacterial and animal strains during research of a specific biological phenomena, and thereby belongs to the history of genetic studies. Furthermore, our herein results on, the SOS response to DNA damage in *E. coli*, reflects the complexity of the interplay between cellular networks, and as such reflects the importance of personalized medicine in general, and specifically in the use of antibiotics due to the expected diversity of individual microbiota.

## Materials and Methods

### Bacterial strains and plasmids

We used the following *E. coli* strains: MC4100*relA* ^+^ (WT) [28] and its derivatives MC4100*relA* ^+^Δ*mazEF* [28], MC4100*relA* ^+^Δ*clpX* [8. We also used strain MG1655 that we have found previously to be defective in EDF production [8]. For testing *E. coli* strains that are commonly used for SOS studies, we used the following strains (obtained from the *E. coli* Stock Center, CGSC, at Yale University in New Haven, Connecticut, USA): AB1932 (*λ^+^, F−, argE3 or argH1, metA28, lacY1 or lacZ4, thi-1, xyl-5 or xyl-7, galK2, tsx-6*) {23], and BW25113 Δ*(araDaraB)567,*Δ*lacZ4787(::rrn3),rph1,*Δ*(rhaDrhaB)568,hsdR514lacI^q^rrnB_T14_*Δ*lacZ_WJ16_hsdR514*Δ*araBAD_AH33_*Δ*rhaBAD_LD78_*), [24], [29]. We constructed *E. coli* MC4100*relA* ^+^ *λ^+^* by its lysogenization from strain MC4100*relA*1*λ^+^*, which was kindly provided by Dr. Ilan Rosenstein. We also constructed *E. coli* strain AB1932*λ*Δ*rexB*, by the use of Datsenko method [31]. For our fluorescence measurements, we used plasmid pL(*lexO*)-*gfp* [22], which carries a *ampR* and thus confers resistance to ampicillin, and which was kindly provided by Dr. Lyle A. Simmons. We constructed plasmid pZE31-*rexB* in which the promoter is inducible by aTc (anhydrotetracycline) [32].

### Materials and Media

We grew the various *E. coli* strains in liquid M9 minimal medium containing 1% glucose and a mixture of 2 mg/ml of each of the essential amino-acids excluding tyrosine and cysteine. Synthetic EDF (NNWNN) and synthetic iEDF (NNGNN) were purchased from GeneScript Corp (Piscataway, NJ, USA).

### Growth conditions

For the experiments in which we measured fluorescence, we diluted over-night cultures harboring plasmid pL(*lexO*)-*gfp* [22] 1∶100 in 10 ml of M9 minimal medium, with 100 µg/ml of ampicillin. We grew the cells at 37°C, with shaking (220 rpm), to O.D_600_ 0.5–0.6. We divided each culture into 500 µl aliquots, and to each aliquot we added the appropriate antibiotics and/or EDF at the concentrations described in the figure legends. These samples were used for fluorescence assays as described below.

### Cell viability

For the experiments on cell viability, we grew cells in 10 ml M9 minimal medium to an optical density at 600 nm (OD_600_) of 0.5 to 0.6. Then, we divided each culture into 500-µl aliquots to which we added the appropriate concentration of NA. We incubated each aliquot at 37°C for 4 h (or as described in the figure legends) and then washed them twice with phosphate-buffered saline (PBS) (pH 7.2). We carried out viability assays on LB plates as we have described previously (6).

### Plaque assay

We grew cells in 10 ml M9 minimal medium with or without antibiotic, as described above, and treated them with appropriate concentration of NA for 4 h (or as described in the figure legends). The supernatants were collected, serially diluted and mixed with soft agar containing TG1 cells. Then, the mixtures were poured on LB agar plates. The plates were incubated at 37°C for overnight and plaque forming units (PFU) were calculated by counting plagues.

### Fluorescence measurements

From the 500 µl aliquot samples of the growing *E. coli* cultures harboring plasmid pL(*lexO*)-*gfp* treated with EDF we placed 250 µl samples into each well of a 96 well plate. In each well, using a 485±15 nm excitation filter and a 530±15 nm emission filter, we measured the fluorescence 25 times at intervals of 10 min. The fluorophore was excited with 1000 CW lamp energy, and the fluorescence in each well was measured for 1 s (FLUOstar galaxy, BMG Labtechnologies).

### Determining the LexA degradation by Western blot analysis

We grew cells in 10 ml M9 minimal medium, as described above, and treated them with appropriate concentration of NA for different periods of time as described in the figure legends. To lyse the cells, we centrifuged samples for 2 min and then suspended the cell pellets in 50 µl of Bugbuster master mix (Novagen), incubating them with vigorous shaking at room temperature for 10 min. Then, we centrifuged them at 4°C for 10 min and transferred the supernatants containing the crude extract to fresh tubes. We determined protein concentrations using the Bradford assay (Bio-Rad, Hercules, CA, USA). For Western blot analysis, we used rabbit polyclonal antibody to the LexA DNA binding region as the primary antibody (Abcam) and donkey polyclonal antibody to rabbit IgG (horseradish peroxidase [HRP]) as the secondary antibody (Abcam).

## Supporting Information

Figure S1 In strain AB1932 the addition of EDF did not inhibit the SOS response. We compared *E. coli* strain MC4100*relA* ^+^ with strain AB1932; it harbored plasmid pL(*lexO*)-*gfp*. We grew the cells as described in the legend to Figure 1. When the culture reached O.D._600_ 0.5–0.6, we added (or not) EDF (10 ng/ml). These cultures were incubated without shaking at 37°C for 30 min, after which we added NA (10µg/ml) to each sample. Immediately after adding NA, we measured fluorescence (FU) by fluorometer over a period of 4 hours. The values shown are relative to those of cells that had not been treated with NA. All data are representative of three independent experiments.
(TIF)

Figure S2 *E. coli* strains commonly used for SOS studies bear the *mazEF* module on their chromosomes. Using two primers, (i) forward primer-GCCGAAATTTGCTCGTATCT and (ii) reverse primer-CTGAAAATTGCGGGTCTGTC, we performed PCR to detect the *mazEF* module in four *E. coli* strains: (1) MC4100*relA* ^+^, (2) MC4100*relA* ^+^Δ*mazEF*, (3) BW25113, (4) AB1932.
(TIF)
