# Supplementary figures and images for: The SOS Response is Permitted in Escherichia coli Strains Deficient in the Expression of the mazEF Pathway
Source: PLoS One. 2014 Dec 3;9(12):e114380. doi: 10.1371/journal.pone.0114380 (PMC4255059; doi:10.1371/journal.pone.0114380)

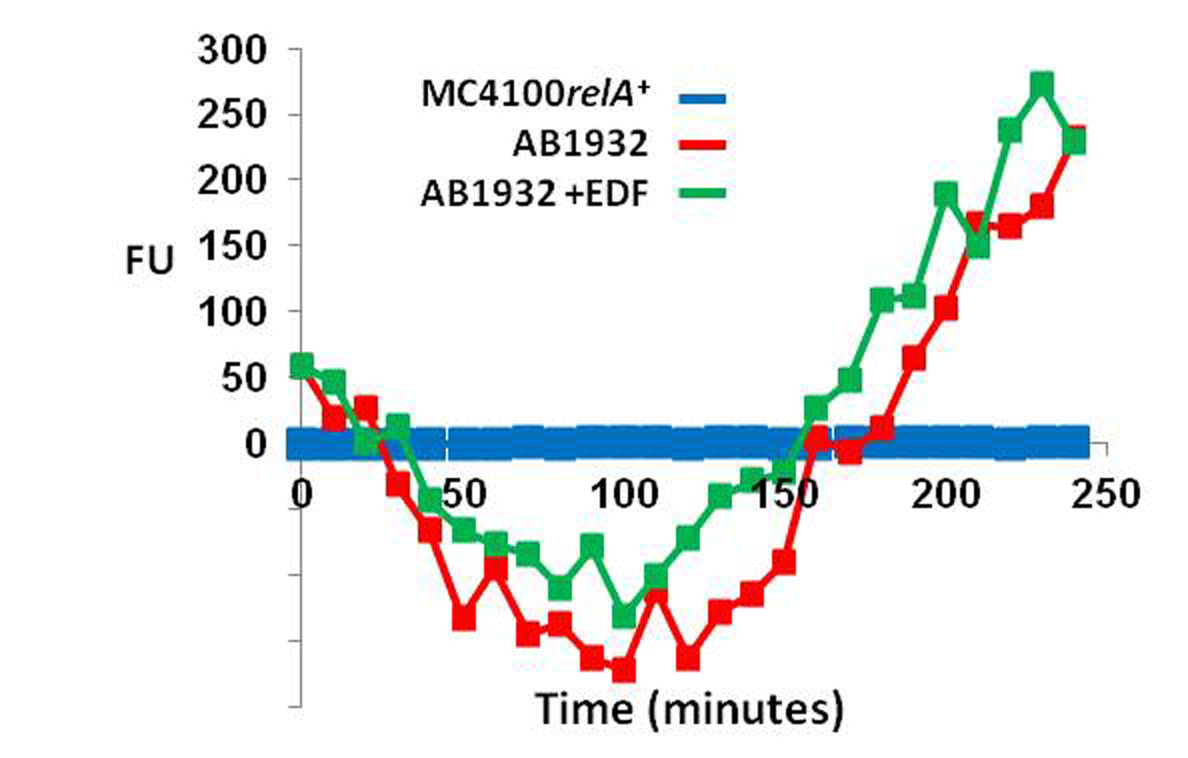

Supplement: Figure S1 — In strain AB1932 the addition of EDF did not inhibit the SOS response. We compared E. coli strain MC4100relA + with strain AB1932; it harbored plasmid pL(lexO)-gfp. We grew the cells as described in the legend to Figure 1. When the culture reached O.D.600 0.5–0.6, we added (or not) EDF (10 ng/ml). These cultures were incubated without shaking at 37°C for 30 min, after which we added NA (10µg/ml) to each sample. Immediately after adding NA, we measured fluorescence (FU) by fluorometer over a period of 4 hours. The values shown are relative to those of cells that had not been treated with NA. All data are representative of three independent experiments. (TIF) [file pone.0114380.s001.tif]

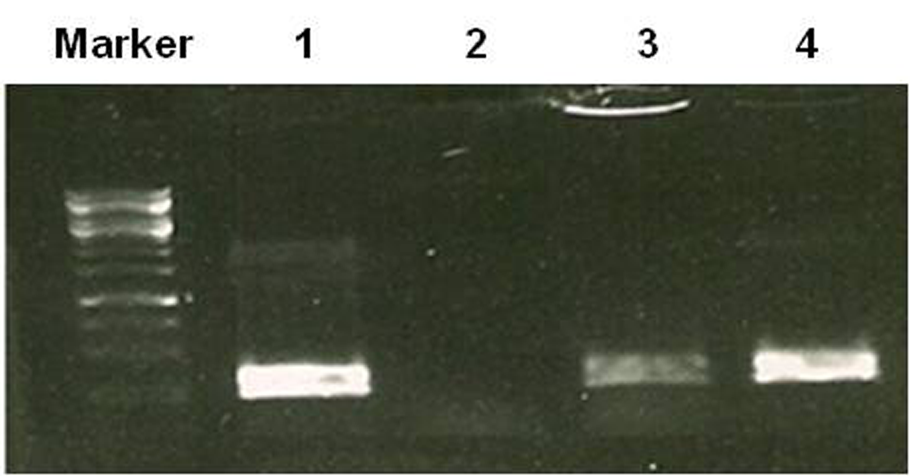

Supplement: Figure S2 — E. coli strains commonly used for SOS studies bear the mazEF module on their chromosomes. Using two primers, (i) forward primer-GCCGAAATTTGCTCGTATCT and (ii) reverse primer-CTGAAAATTGCGGGTCTGTC, we performed PCR to detect the mazEF module in four E. coli strains: (1) MC4100relA +, (2) MC4100relA +ΔmazEF, (3) BW25113, (4) AB1932. (TIF) [file pone.0114380.s002.tif]
